# Organizational support enhances nurses’ work-family enrichment: a person–context interactionist perspective

**DOI:** 10.3389/fpsyt.2024.1392811

**Published:** 2024-05-01

**Authors:** Hao Xu, Xiufang Zhao

**Affiliations:** ^1^Department of Pediatric Intensive Care Unit Nursing, West China Second University Hospital, Sichuan University/West China School of Nursing, Sichuan University, Chengdu, Sichuan, China; ^2^Key Laboratory of Birth Defects and Related Diseases of Women and Children (Sichuan University), Ministry of Education, Chengdu, Sichuan, China

**Keywords:** nurse, organizational support, decent work, burnout, work-family enrichment

## Abstract

**Introduction:**

Attaining a favorable work-life balance is a complex and ongoing challenge in the nursing profession. According to a person–context interactionist perspective and the two-factor theory, this study investigated the underlying mechanism by which organizational support impacts work-family enrichment via protective factors (i.e., decent work) and depleting factors (i.e., burnout) among Chinese nurses.

**Methods:**

A descriptive cross-sectional research design was utilized in this study, employing an online questionnaire as the primary method for data collection. The study included 355 nurses who completed a self-reported questionnaire designed to measure variables such as organizational support, decent work, burnout, work-family enrichment, and demographic information. The collected data were analyzed using a chain mediation model in PROCESS macro (Model 6).

**Results:**

The findings of the analysis revealed that nurses reporting higher levels of organizational support also exhibited a greater sense of work-family enrichment. Moreover, the study identified indirect effects of organizational support on work-family enrichment, mediated by decent work and burnout.

**Discussion:**

These findings suggest that targeted interventions aimed at promoting organizational support can contribute to the overall well-being and work-life balance of nurses.

## 1 Introduction

Work and family play crucial roles in the lives of adults. They are not only fundamental aspects of adulthood but also serve as the primary social function undertaken by individuals. As such, it is not surprising that work and family balance can exert a significant impact on life satisfaction (1, 2). In clinical work, achieving a favorable work-life balance in the nursing profession poses significant challenges (3), especially for female nursing staff (4). The role of nursing in healthcare is pivotal, bearing substantial significance in the holistic advancement of health infrastructure and proactive response to population aging trends. The substantial workload, coupled with insufficient wages and unfavorable working conditions, erodes the drive to effectively juggle nursing duties and family obligations. This further intensifies the already critical shortage of nurses (5). Work–family enrichment (WFE) is one construct representing how work and family benefit each other. The fundamental thinking behind enrichment is that work and family each provide individuals with resources such as enhanced esteem, income, and other benefits that may help the individual better perform across other life domains (6, 7). Earlier research has signaled that WFE is related to numerous factors, such as core self-evaluation, organizational support, flexible work arrangements, turnover intention, job satisfaction, self-efficacy, burnout, and quality of life (8, 9). However, while prior investigations have explored this topic, the existing evidence is preliminary and somewhat inconclusive. More importantly, these findings are fragmented and have not been systematically examined in an integrated model.

Exhilaratingly, the introduction of person-context interaction theory (10) offers a promising framework to effectively address these issues. From the viewpoint of person-context interaction theory (10), human behaviors are influenced by a blend of individual and environmental elements (11). Within this framework, this study explored the effect of external environmental factors (i.e., organizational support) on personal behavior (i.e., WFE) through individual internal factors (i.e., decent work and burnout). This research aimed to provide valuable guidance to nurses, enabling them to develop a positive mindset that allows them to leverage professional advantages and ultimately enjoy the rewards of a harmonious work-family relationship.

### 1.1 Organizational support and work-family enrichment

Organizations are strongly recommended to devote significant attention to the interplay between work and family responsibilities. Indeed, it is in the organization’s best interest to adopt practices that enable nurses to excel in their roles at work while maintaining a meaningful presence within their households (12). Any formal or informal factors that contribute to the realization of work/family balance for nurses are encompassed within the concept of organizational support. According to Halbesleben et al. (13), the term “resources” in the conservation of resources theory is defined as “anything that individuals perceive as capable of helping them achieve their goals”, such as career development opportunities, self-efficacy, job autonomy, etc. Likewise, organizational support can be regarded as a valuable resource that aids nurses in effectively reaching their goals. Empirical studies have reported that WFE is enhanced following an increase in supportive work–family culture (14) and a reduction in organizational work–family barriers (15). According to a survey conducted on 220 employed adults, the analysis of hierarchical regression demonstrated that individuals reported a high level of WFE in highly supportive work environments, irrespective of their core self-evaluations (16). Indeed, when individuals perceive their organization as supportive, they experience a sense of fulfillment in meeting their socioemotional needs within their work role. This fulfillment not only leads to reciprocal positive behaviors at work, such as enhanced performance, but also contributes to a favorable psychological experience that allows individuals to derive benefits from work and apply them to family life (16). Offering nurses Employee Assistance Programs (EAPs), that are employer-sponsored programs designed to support employees in dealing with personal and work-related issues, or counseling services to assist them in coping with personal and family issues (17) can contribute to improved emotional well-being, reducing the negative spillover from one domain to the other and enhancing WFE. Ke et al. (18) discovered that the positive effects of social connectedness on nurses’ life satisfaction were mediated by WFE. Therefore, we anticipate that organizational support can positively predict WFE.

Meanwhile, how does organizational support impact WFE? Social cognitive theory highlights the role of cognitive, environmental, and behavioral factors in shaping human behavior (19). In concrete terms, the environment has the potential to influence specific behaviors by shaping individual psychological perceptions. As a result, based on the two-factor theory, this study examined the effects of organizational support on personal behavior (i.e., WFE) via internal protective factors (i.e., decent work) and depleting factors (i.e., burnout). By delving into these two pathways, we aim to gain a better understanding of the mechanism by which the external environment impacts individual behavior.

### 1.2 The potential mediating effect of decent work

On the one hand, organizational support enhances nurses’ work-family enrichment through increased perceptions of decent work. The notion of decent work, as a global concept, can be traced back to the United Nations Declaration of Human Rights, wherein the importance of work was recognized as an inherent component of human rights (20). It was first defined by the ILO in 1999 as the sum of people’s “aspirations for opportunity and income; rights, voice and recognition; family stability and personal development; and fairness and gender equality” (21). A recent study indicates that the principles associated with decent work may alleviate chronic illness (22). Organizational support encompasses the following aspects: (1) establishing, cultivating, and enhancing social safeguards for employees, such as social security and labor protection, tailored to the specific cultural contexts of societies; (2) affirming, promoting, and ensuring the fundamental rights that underpin a workplace characterized by dignity and fairness and so on (23). As anticipated, all of the aforementioned aspects are related to nurses’ perceived decent work. In the Chinese context, the perception of decent work among nurses is largely influenced by two factors. Firstly, it hinges upon whether the hospital and department adequately meet their basic needs, fostering their willingness to pursue their nursing career. Secondly, it is shaped by the opinions of those in their social circle, particularly families and friends, regarding the nursing profession. Evidently, these opinions are heavily influenced by the hospital’s attitude and practices towards nurses, encompassing aspects such as welfare benefits and career development. In fact, the Chinese conventional concept of valuing medical treatments over nursing care lowers the social status of nurses (5). Therefore, organizational support plays a vital role in empowering nurses to perceive their work as dignified labor. Extensive psychological research has meticulously outlined the multifaceted pathways through which access to employment enhances psychological well-being (24, 25). Concrete evidence underscored that offering access to decent work serves as a catalyst for fostering WFE within public and private universities in North India (26). Therefore, it is reasonable to speculate that organizational support provides nurses with a sense of decency, thus enabling them to leverage the mutual benefits of work and family.

### 1.3 The potential mediating effect of burnout

On the other hand, organizational support enhanced nurses’ work-family enrichment through reduced burnout. According to the Job Demands-Resources (JD-R) model introduced by Demerouti et al. (27), job-related factors can be classified into two categories: job demands and job resources. When an individual is unable to satisfy job requirements over an extended period from both organizational and societal perspectives, it can lead to feelings of job burnout, which in turn affect job performance. Burnout is a condition of both physical and mental exhaustion that arises due to prolonged and persistent stress stemming from work-related factors (28). It is important to recognize and address occupational burnout to maintain overall well-being and prevent its detrimental effects on both individuals and organizations (29, 30). Additionally, burnout can result from various factors, including excessive work demands, lack of control or autonomy, inadequate support, and a mismatch between an individual’s values and the organizational culture. However, if organizations can provide physical or psychological support, nurses are less likely to experience burnout. Li et al. (31) noted that perceived organizational support significantly affected nurses’ intention to stay. Burnout carries significant repercussions, detrimentally impacting individuals’ functioning (28). It can lead to reduced performance, motivation, and overall effectiveness while also curtailing opportunities for fulfilling work experiences and organizational commitment (28, 32). Burnout, as an occupational hazard, impacts not only nurses but also patients, organizations, and society as a whole. It is associated with a deterioration in safety and care quality, decreased patient satisfaction, and a reduction in nurses’ organizational commitment and productivity (33). Consequently, nurses with high levels of burnout face challenges both in their professional and personal families, as these two domains cannot mutually benefit each other.

Given that burnout yields adverse consequences for both individuals and organizations (34), and considering that the work environment stands as a crucial predictor of this syndrome, it becomes pertinent to explore this phenomenon within the context of a precursor such as the absence of decent work. Previous research has unveiled a noteworthy relationship between the perception of decent work and the occurrence of burnout (35, 36). For instance, Ferraro et al. (35) delved into the interplay between decent work, work engagement, work motivation, and physician burnout and showcased that decent work correlates with elevated levels of autonomous work motivation, leading to increased work engagement and lower personal burnout. Consequently, the present study adopted a chain mediation model to further investigate these dynamics.

### 1.4 Aims of the present study

**Figure 1:**
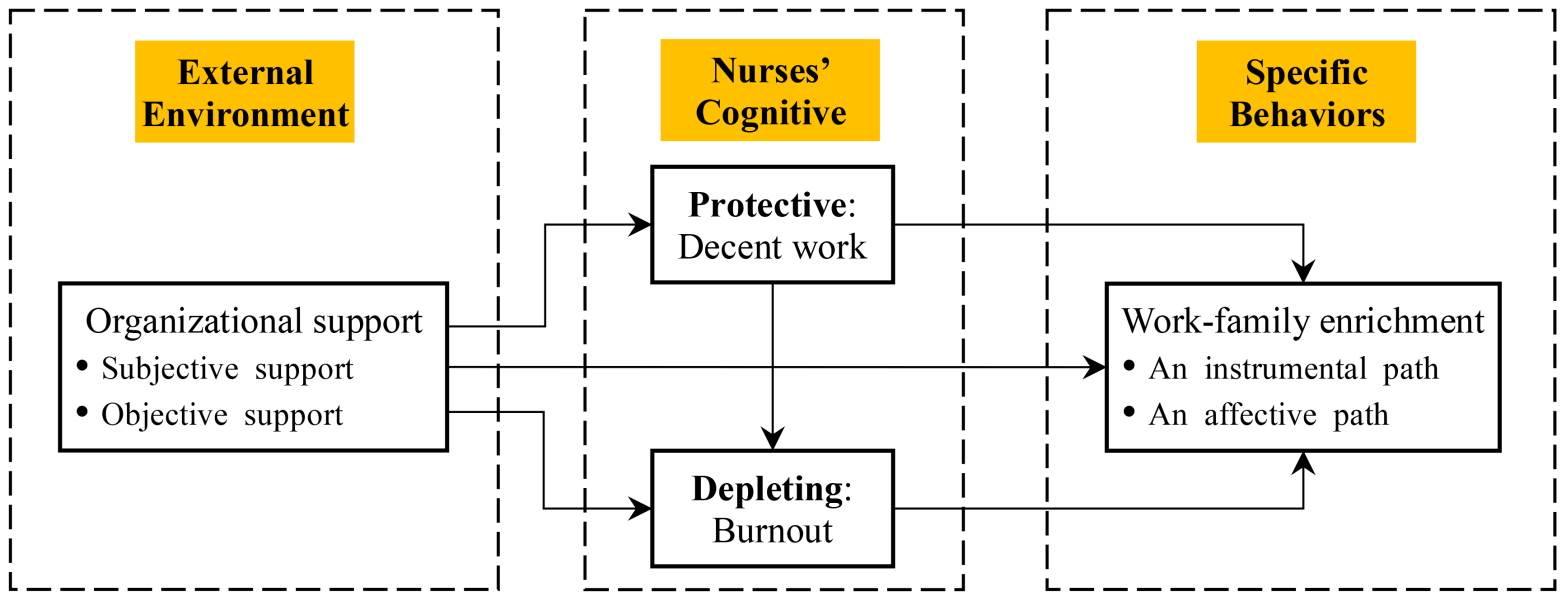
Hypothetical model of work-family enrichment among nurses.

While several studies have affirmed the connection between decent work, burnout, organizational support, and WFE, the precise nature of their interrelationships remains somewhat elusive. Thus, this study aimed to investigate the potential mediating roles of both decent work and burnout in this complex interplay. By exploring the intricate connections between these four variables, this research seeks to establish a comprehensive theoretical framework that contributes to the advancement of WFE practices, specifically within the context of Chinese nurses. Based on prior empirical investigations, a theoretical model was formulated to underpin our hypotheses. We posit the following four hypotheses: (1) Positive prediction of WFE: Organizational support is expected to exert a positive predictive effect on the WFE of nurses; (2) Mediating role of decent work: The impact of organizational support on nurses’ WFE is anticipated to be partially mediated by the level of decent work experienced by nurses; (3) Mediating role of burnout: Organizational support’s influence on WFE is projected to be partially mediated by the level of burnout experienced by nurses; (4) Chain mediation through decent work and burnout: The predictive effect of organizational support on nurses’ WFE is expected to be indirectly mediated through a chain of mediating variables, involving both decent work and burnout. The hypothetical model is illustrated in **Figure 1**. By systematically examining these hypotheses, we expect to offer robust empirical backing for the person-context interaction theory while also contributing valuable insights to the ongoing reform of Chinese health care system.

## 2 Method

### 2.1 Participants and procedure

A web-based questionnaire platform, Wenjuanxing, was utilized to conduct a survey targeting nurses employed in hospitals located in Sichuan Province. The purpose of this research was initially explained to the nursing managers of the hospitals. Upon obtaining informed consent, the QR code or questionnaire link was distributed to the respective nursing workgroups, allowing for anonymous completion of the survey. The questionnaire was composed of fundamental demographic information and relevant scales specifically designed to mandate responses for all questions and prevent duplicate answers. Gratitude was expressed upon completion of the questionnaire. The inclusion criteria for this study were as follows: (1) actively working nurses who possessed and registered their nursing qualifications, (2) a minimum work experience of 1 year, and (3) voluntary participation in the study. The study excluded individuals who met the following criteria: (1) nurses engaged in external training, residency programs, or internships, (2) nurses on maternity leave, sick leave, or other types of absence during the survey period, and (3) nurses in non-clinical positions. A total of 380 questionnaires were distributed for this study, with 355 questionnaires being collected, resulting in an effective response rate of 93.42%. In this study, there were 320 females (90.14%) and 35 males (9.86%). In most countries, there are far more women than men in the nursing profession (37). According to Chinese National Health Commission, male nurses constituted 3 percent of the total number of nurses, which exceeds 5 million in the country by the end of 2021. The age ranged from 21 to 58 years (*M* = 31.04, *SD* = 0.35). Regarding educational attainment, 6 participants had completed vocational school (1.69%), 80 had completed college (22.54%), 264 had a bachelor’s degree (74.37%), and 5 had a master’s degree or higher (1.41%). There were 225 married participants (63.4%) and 130 unmarried participants (36.6%). In terms of monthly income, 26 participants earned less than 413.76 $ (7.3%), 91 earned between 413.90 and 689.61 $ (25.6%), 192 earned between 689.75 and 1379.22 $ (54.1%), and 46 earned more than 1379.22 $ (13.0%). The years of work experience ranged from 1 to 37 years (*M* = 8.97, SD = 7.21). The categories of nurses encompassed in our survey encompassed a wide range, including pediatric nurses, obstetric nurses, gynecological nurses, medical nurses, surgical nurses, and other relevant specialties. Sensitivity analysis using G*Power (38) was conducted to determine that the sample size was capable of detecting an effect size of *f* ^2 = ^0.021 at α = 0.05 and power = 0.80. This study was approved by the Institutional Review Board of the corresponding author’s institution.

### 2.2 Measures

#### 2.2.1 Organizational support

The Perceived Organizational Support Scale, comprising 8 items (39), was employed to assess nurses’ perceptions of the support offered by their organization. Sample items within the scale include statements such as “The organization genuinely cares about my well-being”. Respondents rated these items on a 5-point scale, ranging from 1 (strongly disagree) to 5 (strongly agree). The resulting scores were acquired by calculating the mean of participants’ responses, with higher scores reflecting a higher perceived level of organizational support. The utilization of confirmatory factor analysis yielded robust results, affirming the scale’s fit within the dataset (χ^2^/*df* = 5.63, CFI = 0.98, TLI = 0.96, RMSEA = 0.11, 90%CI [0.08, 0.15], and SRMR = 0.02), indicative of its strong construct validity. Furthermore, the internal consistency of the scale was demonstrated by a Cronbach’s alpha coefficient of 0.94, implying a high level of reliability. This was further corroborated by the McDonald’s ω coefficient, which also stood at 0.94, further attesting to the scale’s dependability.

#### 2.2.2 Decent work

Nurses’ perception of decent work was assessed using the 16-item Perceived Decent Work Scale designed by Mao and Liu (40). This scale encompasses five distinct dimensions: job rewards, job position, career development, career recognition, and working atmosphere. Sample items from the scale include statements like “The hospital provides me with commendable benefits (such as paid leave, holiday bonuses, etc.)”. Respondents indicated their agreement with these items on a 5-point scale, ranging from 1 (extremely disagree) to 5 (extremely agree). The scores were computed by averaging participants’ responses, with higher scores denoting a stronger perception of decent work. The application of confirmatory factor analysis yielded a satisfactory fit for the scale within the dataset (χ^2^/*df* = 2.03, CFI = 0.95, TLI = 0.94, RMSEA = 0.05, 90%CI [0.04, 0.06], and SRMR = 0.06), corroborating its robust construct validity. Moreover, the scale demonstrated good internal consistency, evident from a Cronbach’s alpha coefficient of 0.86, signifying reliability. This was further affirmed by the McDonald’s ω coefficient, which stood at 0.87, reinforcing the scale’s reliability.

#### 2.2.3 Burnout

Burnout was assessed using the Maslach Burnout Inventory – General Survey (41), a well-established measure in the field. This survey is composed of 16 items that are organized into three distinct subscales. The first subscale, labeled “exhaustion”, comprises five items and evaluates feelings of burnout, such as the statement “I feel burned out from my work”. The second subscale, termed “cynicism”, consists of five items, including an example statement like “I have become less enthusiastic about my work”. The remaining six items form the third subscale, which assesses the dimension of professional efficacy. An example statement from this subscale is, “I feel confident that I am effective at getting things done”. Respondents rate each item on a seven-point Likert scale, ranging from 0 (“never”) to 6 (“every day”). To calculate scores, individual responses are averaged. Higher scores on the scale reflect a higher level of job-related burnout. The validity of the scale was established through confirmatory factor analysis, revealing favorable fit indices (χ^2^/*df* = 4.45, CFI = 0.93, TLI = 0.92, RMSEA = 0.10, 90%CI [0.09, 0.11], and SRMR = 0.06), suggesting strong construct validity. Internal consistency reliability was confirmed with a Cronbach’s alpha value of 0.91 and a McDonald’s ω value of 0.92, both signifying strong internal cohesion within the scale.

#### 2.2.4 Work-family enrichment

The 14-item Work-Family Enrichment Scale (6, 42) was utilized to evaluate the extent of work-family enrichment experienced by nurses. The scale consists of sample statements such as “Working taught me to listen and understand points of view that are different from my own and helped me perform better with my family”. Participants rated these statements on a 5-point scale, ranging from 1 (“extremely disagree”) to 5 (“extremely agree”). To compute scores, responses were averaged, with higher scores suggestive of more work-family enrichment. The validity of the scale was established through confirmatory factor analysis, yielding favorable fit indices (χ^2^/*df* = 4.58, CFI = 0.94, TLI = 0.93, RMSEA = 0.10, 90%CI [0.09, 0.11], and SRMR = 0.05), suggesting robust construct validity. The internal consistency reliability was verified, as evidenced by a Cronbach’s alpha coefficient of 0.95 and a McDonald’s ω coefficient of 0.95. These results emphasize the high internal consistency present within the scale.

### 2.3 Data analysis

Statistical analyses were performed using SPSS 24.0 software. Initially, descriptive statistics were conducted on both demographic variables and four key factors. To assess our hypotheses, Pearson correlation analysis was utilized to investigate the connections between organizational support, decent work, burnout, and WFE. Subsequently, the SPSS PROCESS macro 3.3 software, developed by Hayes (43), was applied to probe the potential mediating roles of both decent work and burnout in the link between organizational support and WFE. This software was specifically tailored for examining intricate models. Within the PROCESS framework, model 6 was used, focusing on two mediators. To gauge indirect effects, a bias-corrected bootstrapping procedure was employed. Notably, 95% confidence intervals (CI) not including 0 indicated a significant mediation effect. Covariates such as gender, age, education, marital status, monthly income, and working years within the model were adjusted for.

## 3 Results

### 3.1 Common method biases

In order to address concerns about common method biases (CMB) in the questionnaire survey following Podsakoff et al.’s approach (44), Harman’s single-factor test was carried out to evaluate the presence of CMB within our collected data. The outcomes of this test, based on exploratory factor analysis without rotation, revealed the extraction of 10 factors with eigenvalues exceeding 1. However, the highest factor variance explained was 35.20%, falling below the critical threshold of 40% suggested by Podsakoff et al. (44). Furthermore, the adequacy of the single-factor model fit was found to be unsatisfactory, as indicated by the following fit indices: χ^2^/*df* = 7.34, CFI = 0.46, TIL = 0.44, RMSEA = 0.13, and SRMR = 0.12. In contrast, the fit indices of the four-factor model were deemed acceptable: χ^2^/*df* = 5.77, CFI = 0.90, TIL = 0.85, RMSEA = 0.10, and SRMR = 0.07. These outcomes collectively indicated that CMB was not significant within our dataset. While it was not possible to completely eliminate the potential contamination issue caused by CMB, these outcomes can enhance our confidence in the findings.

### 3.2 Descriptive statistics

Means, standard deviations, and correlations are detailed in **Table 1**. Organizational support, decent work, and burnout were all positively correlated with WFE (*r* = 0.50, *p* < 0.001; *r* = 0.55, *p* < 0.001; and *r* = -0.65, *p* < 0.001, respectively). Furthermore, organizational support, decent work, and job burnout were significantly and positively correlated with each other. The correlations among the study variables offer preliminary support for our mediation model. Of note, previous studies have documented that WFE may be affected by gender, marital status, and work hours (45). In addition, this study observed that age, marital status, monthly income, and working years were significantly correlated with WFE. Therefore, in this study, these demographic variables were incorporated into the model as control variables.

**Table 1:** Descriptive statistics and correlations for all variables.

	*M*	*SD*	1	2	3	4	5	6	7	8	9	10
1 Gender	0.10	0.30	1									
2 Age	31.04	6.65	-0.14^**^	1								
3 Education	2.75	0.50	0.11^*^	0.22^***^	1							
4 Marital status	0.37	0.48	0.04	-0.54^***^	-0.23^***^	1						
5 Monthly income	2.73	0.78	0.03	0.43^***^	0.41^***^	-0.30^***^	1					
6 Working years	8.97	7.21	-0.15^**^	0.91^***^	0.14^**^	-0.51^***^	0.38^***^	1				
7 Organizational support	3.01	0.77	0.04	0.11^*^	0.02	-0.06	0.18^**^	0.08	1			
8 Decent work	3.03	0.54	-0.02	0.17^**^	0.03	-0.10	0.30^***^	0.15^**^	0.69^***^	1		
9 Burnout	2.22	0.96	0.02	-0.25^***^	-0.03	0.16^**^	-0.16^**^	-0.24^***^	-0.48^***^	-0.58^***^	1	
10 WFE	3.51	0.76	0.03	0.24^***^	0.07	-0.24^***^	0.15^**^	0.22^***^	0.50^***^	0.55^***^	-0.65^***^	1

^∗^ p < 0.05, ^∗∗^ p < 0.01, ^∗∗∗^ p < 0.001. Gender: 0 = female; 1 = male. Marital status: 0 = married, 1 = unmarried. WFE, Work-family enrichment.

### 3.3 The chain mediating effects analyses

To begin, the relationship between organizational support and WFE was examined. The results signaled that organizational support positively predicted WFE after controlling for gender, age, education, marital status, monthly income, and working years, *B* = 0.49, SE = 0.05, *p* < 0.001, 95%CI[0.38, 0.59], adjusted *R*^2 = ^0.28, *F* (6, 348) = 23.87, *p* < 0.001.

**Figure 2:**
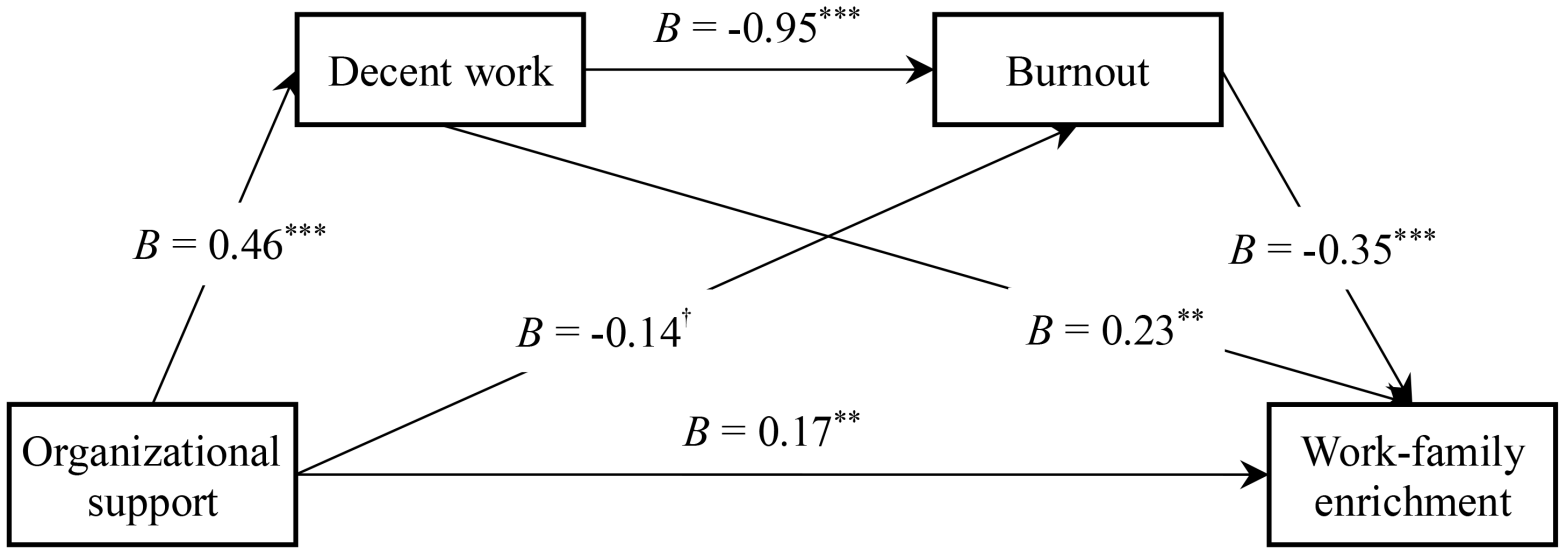
The mediating effect of decent work and burnout in the association between organizational support and work-family enrichment. Unstandardized regression coefficients are presented. ^†^ *p* < 0.1, ^∗∗^ *p* < 0.01, ^∗∗∗^ *p* < 0.001.

Next, Model 6 in the SPSS macro PROCESS developed by Hayes (43) and the bias-corrected Bootstrap (sample size 5000) method were used to test the chain mediation effect. With organizational support as the independent variable, WFE as the dependent variable, decent work and burnout as chain mediating variables, and gender, age, education, marital status, monthly income, and working years as control variables, the path coefficient results are summarized in **Table 2** and **Figure 2**. The overall regression equation was significant, with adjusted *R*^2^ = 0.50, MSE = 0.29, *F* (9, 345) = 39.07, *p* < 0.001. Importantly, organizational support was a significant predictor of decent work (*B* = 0.46, SE = 0.03, *t* = 17.37, *p* < 0.001, 95%CI [0.41, 0.52]), organizational support exhibited a marginal predictive impact on burnout (*B* = -0.14, SE = 0.07, *t* = -1.89, *p* = 0.060, 95%CI [-0.28, 0.00]), decent work demonstrated a notable predictive impact on burnout (*B* = -0.95, SE = 0.11, *t* = -9.05, *p* < 0.001, 95%CI [-1.17, -0.75]), organizational support emerged as a significant predictor of WFE (*B* = 0.17, SE = 0.05, *t* = 3.20, *p* < 0.01, 95%CI [0.07, 0.28]), decent work had a substantial predictive influence on WFE (*B* = 0.23, SE = 0.09, *t* = 2.60, *p* < 0.01, 95%CI [0.05, 0.40]), and burnout emerged as a significant predictor affecting WFE (*B* = -0.35, SE = 0.04, *t* = -9.03, *p* < 0.001, 95%CI [-0.43, -0.28]). The path indirect effect with decent work as the mediating variable was 0.11 (95%CI [0.02, 0.19]), whilst the path indirect effect with burnout as the mediating variable was 0.05 (95%CI [0.01, 0.10]). Contrastingly, the path indirect effect of decent work and burnout as mediating variables was 0.16 (95%CI [0.11, 0.23]), whereas the total indirect effect was 0.31 (95%CI [0.22, 0.40]) (see **Table 3**). Taken together, these results established the role of decent work and burnout in the positive effect of organizational support on WFE.

**Table 2:** Regression analysis of the relationship between organizational support and work-family enrichment.

	Decent work		Burnout		Work-family enrichment	
	*B*	95%CI	*B*	95%CI	*B*	95%CI
Gender	-0.08	[-0.21, 0.05]	-0.07	[-0.34, 0.20]	0.13	[-0.07, 0.33]
Age	-0.01	[-0.02, 0.01]	-0.01	[-0.04, 0.02]	0.01	[-0.02, 0.03]
Education	-0.07	[-0.16, 0.01]	-0.04	[-0.21, 0.14]	0.08	[-0.05, 0.21]
Marital status	0.01	[-0.09, 0.10]	0.07	[-0.13, 0.26]	-0.23^**^	[-0.37, -0.09]
Monthly income	0.14	[0.08, 0.20]	0.14^*^	[0.02, 0.26]	-0.06	[-0.16, 0.03]
Years of work	0.01	[-0.01, 0.02]	-0.02	[-0.04, 0.01]	0.01	[-0.01, 0.03]
Organizational support	0.46^***^	[0.41, 0.52]	-0.14^†^	[-0.28, 0.00]	0.17^**^	[0.07, 0.28]
Decent work			-0.95^***^	[-1.17, -0.75]	0.23^**^	[0.05, 0.40]
Burnout					-0.35^***^	[-0.44, -0.28]
*R*^2^	0.52 63.09		0.41 34.66		0.50 39.07	
*F*						

^†^ p < 0.1, ^∗^ p < 0.05, ^∗∗^ p < 0.01, ^∗∗∗^ p < 0.001.

**Table 3:** Chain mediating effects of decent work and burnout.

	Direct effects			Indirect effects		
	Effect	SE	95%CI	Effect	SE	95%CI
Model 1: X → Y	0.17	0.05	[0.07, 0.28]	0.31	0.05	[0.23, 0.41]
Model 2: X → M1 → Y				0.11	0.04	[0.02, 0.19]
Model 3: X → M2 → Y				0.05	0.03	[0.01, 0.10]
Model 4: X → M1 → M2 → Y				0.16	0.03	[0.11, 0.23]

X, Organizational support; Y, Work-family enrichment; M1, Decent work; M2, Burnout.

## 4 Discussion

This study adopted a chain mediation model to analyze the intricate connection between organizational support and WFE within the context of currently employed nurses in China. Noteworthily, the findings validate a direct correlation between organizational support and WFE among nurses, aligning with previous research by Ghislieri et al. (46) and Rashid et al. (12). The outcomes of the analysis revealed that the influence of organizational support on WFE operates through three distinct pathways: decent work, burnout, and the interplay between decent work and burnout. These insights yield a deeper comprehension of the intricate relationship between organizational support and WFE. Importantly, they also offer valuable guidance to relevant institutions, enabling them to implement measures that foster and enhance WFE among Chinese nurses.

The ecological systems theory emphasizes that the development of individuals is nested within a series of interrelated environmental systems (47). For nurses, the hospital can be an ecosystem, and they can be deeply affected by the hospital. In a cross-sectional study conducted amidst the Coronavirus Disease 2019 pandemic, family support and job support were found to play significant roles in predicting the elevated levels of WFE among Chinese nurses who were actively involved in Wuhan. The research, led by Y. Zhang et al. (48), demonstrated the pivotal contribution of organizational support in facilitating nurses to effectively manage the delicate equilibrium between their professional responsibilities and family commitments.

### 4.1 The mediating role of decent work

Herein, the mediating role of decent work in the relationship between organizational support and WFE was established. This observation is in line with the observations of prior research, which indicated that the perception of organizational support positively predicts individuals’ self-efficacy in effectively navigating their professional and personal spheres, consequently resulting in WFE, as well as family-to-work enrichment (49). The significant correlation between higher support for employees and their positive perception of the work environment has become a universally acknowledged consensus. The provision of higher levels of support to nurses has been found to be conducive to their enhanced sense of decent work, encompassing aspects such as job security, fair compensation, and opportunities for professional growth (50). Furthermore, our findings exposed that this enhanced sense of decent work among nurses is positively related to a greater degree of WFE (26). As previously mentioned, WFE refers to the extent to which experiences and resources from the work domain positively affect the family domain, leading to improved well-being and satisfaction in both areas of life (7). The positive correlation between a sense of decent work and WFE portrays the importance of creating supportive work atmosphere that encourage nurses to balance their work and family responsibilities. Hospital managers are encouraged to prioritize the comprehensive well-being of nurses, addressing both their physical and psychological needs, such as expanding career advancement pathways and enhancing compensation levels, among other initiatives. Providing optimal support from the hospital can aid nurses in developing a strong sense of professional worth and purpose, thereby converting work-related fulfillment into beneficial support for their families.

### 4.2 The mediating role of burnout

Our study also documented the mediating role of burnout in the relationship between organizational support and WFE. In the present study, increased support for nurses was associated with lower levels of burnout, which, in turn, positively influenced WFE. The surge in workloads, coupled with a distinct absence of necessary resources and proper support, ranks among the leading contributors to burnout (51). On the other hand, organizational support, such as the provision of resources, recognition, and flexible work arrangements, has been shown to mitigate burnout among nurses (52). Dall’Ora et al. (53) discovered that unfavorable job characteristics such as high workload, inadequate staffing levels, extended shifts, and limited control are linked to nursing burnout. By resolving factors contributing to burnout, organizations can create a more supportive work environment that promotes nurses’ well-being and fosters a better work-family balance. Moreover, our findings insinuate that reducing burnout can enhance the positive relationship between organizational support and WFE. Indeed, nurses more likely to benefit from the support provided by the organization experience lower levels of burnout, leading to greater WFE. These findings emphasize the importance of addressing burnout as a crucial factor in promoting WFE among employees (54). Hospital and nursing managers ought to formulate strategies aimed at mitigating nurses’ burnout and improving their overall quality of work life (55). For example, the introduction of an employee care program, coupled with organizing themed seminars and professional training, as well as alleviating the stresses of work and personal life, all contribute to the establishment of a healthy and well-structured work environment.

### 4.3 The chain mediating role of decent work and burnout

Additionally, our study uncovered a noteworthy pathway denoting the sequence of organizational support → decent work → burnout, and, subsequently, WFE. This model accentuates the intricate connection wherein the mediating role of the chain relationship between decent work and burnout serves to bridge the association between organizational support and the experience of WFE. According to this model, organizational support positively influences nurses’ perception of decent work, which, in turn, leads to lower levels of burnout. Decent work factors, such as job security, autonomy, and supportive relationships (20, 23), enhance the work environment and limit the risk of burnout among nurses. In turn, decreased burnout levels have a positive impact on WFE, considering that nurses experience less emotional exhaustion and possess greater resources to engage in their family roles. By considering the chain relationship between organizational support, decent work, burnout, and WFE, healthcare organizations can implement targeted interventions to enhance work-family balance among nurses. These strategies may involve promoting organizational support, developing policies to enhance decent work conditions, and implementing initiatives to prevent and manage burnout. The conclusion is also consistent with the perspective of person-context interaction theory (10) that highlight the interactive processes through which personal characteristics and context influence each other, ultimately shaping individuals’ experiences, behaviors, and outcomes. Overall, this study theoretically establishes that the psychological and behavioral aspects of nurses are not limited to individual concerns; they are also significantly shaped by external environmental influences. This holds a meaningful source of inspiration for the field of nursing psychology research.

### 4.4 Implication and limitations

Within the framework of person-context interaction theory, this study explored the effect of organizational support on personal behavior (i.e., WFE) through internal protective factors (i.e., decent work) and depleting factors (i.e., burnout). The research findings have significant implications for guiding nurses’ career development and psychological well-being.

Nurses hold a vital position within hospitals, drawing substantial attention and care from various quarters of society, owing to the profound importance of safeguarding their physical and mental health (56). Additionally, nursing is a demanding profession, frequently necessitating nurses to prioritize the needs of patients and families over their own (57). Furthermore, the escalating strain in doctor-patient relationships has led to a significant increase in the incidence of violence against medical professionals, emerging as a prominent social issue in China. Nurses, often positioned as intermediaries between patients and doctors, unfortunately, also fall victim to this violence (5). These issues collectively constitute the unmet focal points in nursing, which are critical to steering healthcare reform in China toward a more constructive trajectory. Addressing those challenges warrants a multifaceted approach, encompassing an increase in nurse training programs, enhancements in income structures, amelioration of working conditions (5). The evidence provided by this study offers invaluable insights and direction for the formulation of effective hospital management strategies. Hospital administrators should prioritize providing comprehensive support to nurses, both in their professional and personal lives, while also striving to lighten their work-related burdens. This approach can effectively minimize conflicts and enhance their overall sense of enrichment. Of note, nurses who have dependent children and work in in-patient settings have expressed a clear need for increased flexibility in their work conditions to achieve a better work-life balance (58). By diligently implementing these strategic measures, hospitals can actively promote a healthier equilibrium between work and personal life for their nursing staff. In doing so, they contribute not only to the nurses’ well-being but also to the overall effectiveness of patient care.

This study has limitations that should be acknowledged. To begin, the cross-sectional design limited our ability to establish causal relationships, exclusively offering a partial glimpse into correlations among variables. Additionally, the dynamic nature of WFE, which can vary in conflict and across different timeframes, was not comprehensively considered (59). As a result, future investigations could adopt longitudinal tracking or log-based research methodologies to uncover the causal intricacies of these variables, thereby offering a more comprehensive and lucid understanding of the mechanisms underlying WFE. Secondly, the reliance on self-reported questionnaires introduces potential biases stemming from social desirability and self-presentation, given the inclusion of sensitive private details. Subjective judgments have the potential to influence both the credibility and generalizability of our findings. To mitigate this, future studies could explore alternative sources of assessment, such as evaluations from educational institutions or family members. Furthermore, the study’s sampling was restricted to nurses in Sichuan Province, which the generalizability of the findings. Expanding the scope of investigation in future studies could enhance the breadth and applicability of the results. Lastly, while this study explored mediating relationships among variables, it did not extensively analyze the potential influence of moderating variables, such as hospital type (public, private, joint venture), on WFE. Certainly, the psychological experiences of nurses could potentially vary among different healthcare institutions owing to the distinct disparities in their respective work environments, especially when there is inconsistency between the actual support provided by the hospital and the support perceived by nurses themselves.

## 5 Conclusion

Despite the aforementioned limitations, this study thoroughly investigated the intricate dynamics between organizational support, decent work, burnout, and WFE among nurses, employing a serial mediating model. Through this exploration, the study shed light on the instrumental role of decent work as a mediator, facilitating the link between organizational support and WFE within the nursing domain. Moreover, the study underscored the mediating influence of burnout within the nursing work environment. Notably, it exposed the consequential chain mediation mechanism, wherein decent work and burnout jointly mediate the connection between organizational support and WFE. In practical, hospitals must continue to enhance their investments to fulfill the diverse needs of nurses comprehensively. This proactive approach ensures that nurses perceive the hospital’s concern and compassion, fostering a conducive environment for their wholehearted dedication to their professional duties. Such measures are not only advantageous for the well-being of nurses but also contribute significantly to the psychological well-being and morale of the entire hospital staff.

## Data availability statement

The raw data supporting the conclusions of this article will be made available by the authors, without undue reservation.

## Ethics statement

The studies involving humans were approved by West China Second University Hospital, Sichuan University. The studies were conducted in accordance with the local legislation and institutional requirements. The participants provided their written informed consent to participate in this study.

## Author contributions

HX: Writing – original draft. XZ: Writing – review & editing, Supervision.
